# A New European Neglected Diseases Center for Greece?

**DOI:** 10.1371/journal.pntd.0001757

**Published:** 2013-02-28

**Authors:** Peter J. Hotez, T. Dorina Papageorgiou

**Affiliations:** 1 Departments of Pediatrics and Molecular Virology & Microbiology, and National School of Tropical Medicine, Baylor College of Medicine, Houston, Texas, United States of America; 2 Sabin Vaccine Institute and Texas Children's Hospital Center for Vaccine Development, Houston, Texas, United States of America; 3 James A. Baker III Institute for Public Policy, Rice University, Houston, Texas, United States of America; 4 Department of Neuroscience, Baylor College of Medicine, Houston, Texas, United States of America

*Following the recently evolving downturn in the Greek economy, there is an opportunity to build a new Hellenic scientific institution for neglected infections of poverty located at the geographic center of Europe, the Middle East, and North Africa.*

While today populations who live in poverty in sub-Saharan Africa and Southeast Asia suffer from the largest public health impact from the world's neglected tropical diseases (NTDs) [1], [2], it is astonishing to some that many of these same diseases also disproportionately strike the impoverished populations living in Europe, the Middle East, and North Africa.

In Europe, a 2011 analysis found surprisingly high rates of NTDs among the more than 150 million Europeans who live below the poverty level [3]. Especially in the Balkans and elsewhere in southeastern Europe, as well as in Turkey, several former Soviet-bloc countries, and among the Roma, today there are high rates of parasitic infections such as echinococcosis, toxocariasis, toxoplasmosis, and trichinellosis, as well as a number of important bacterial infections such as brucellosis and congenital syphilis (Table 1). Many of these NTDs are zoonoses transmitted from animals and linked to breakdowns in veterinary public health, especially in Eastern Europe [3]. Fallout from the war in the Balkans and the collapse of communism among the former Soviet republics represent key socioeconomic determinants of these infections [3].

**Table 1:** Major NTDs of Europe and the Middle East and North Africa.

NTDs	Europe	Middle East & North Africa
***Parasitic Infections***		
Ascariasis/trichuriasis	+	+
Toxocariasis	+	+
Fascioliasis	+	+
Schistosomiasis	−	+
Taeniasis	+	+
Trichinellosis	+	−
Echinococcosis	+	+
Leishmaniasis	+	+
Toxoplasmosis	+	+
Vivax malaria	+	+
***Bacterial/Viral Infections***		
Brucellosis	+	+
Leptospirosis	+	+
Chikungunya virus	+	+
Crimean Congo haemorrhagic fever	+	+
Dengue	+	+
Rift Valley fever	−	+

Similarly, in the Middle East and North Africa, high rates of some of these same parasitic infections are endemic, in addition to fascioliasis, leishmaniasis, and schistosomiasis [4]. Important viral infections have also emerged, including dengue and Rift Valley fevers [4]. Many of these NTDs are also zoonoses transmitted from livestock and other domestic animals and are on the rise as a result of breakdowns in veterinary health and unrestricted trafficking of animals across unsupervised borders [4]. Most of these infections are concentrated among the more than 60 million people who live in the Middle East and North Africa on less than US$2 per day [4]. In terms of the largest number of cases of these conditions, destabilized countries such as Egypt, Iraq, Syria, and Yemen suffer the most from NTDs [4].

The NTDs are forgotten diseases that disproportionately afflict the forgotten poor. Indeed, these neglected infections can even trap the poor in a cycle of poverty because of their ability to stunt child growth and intellectual development, affect pregnancy outcome, and reduce productivity [1]. While the world's attention appropriately turns to completing the Millennium Development Goals by lifting the poorest people in sub-Saharan Africa and Asia out of poverty, we must not leave behind the poorest and forgotten people of Europe, the Middle East, and North Africa.

A key step to addressing these neglected infections of poverty would include establishing a new center for research in order to conduct fundamental and translational research on the major NTDs affecting areas of poverty in southeastern Europe, the Middle East, and North Africa; ideally, these activities might also include product development activities for a new generation of neglected disease drugs, diagnostics, and vaccines. In some cases, new products for veterinary use must be developed as new tools to halt zoonotic transmission.

**Figure 1:**
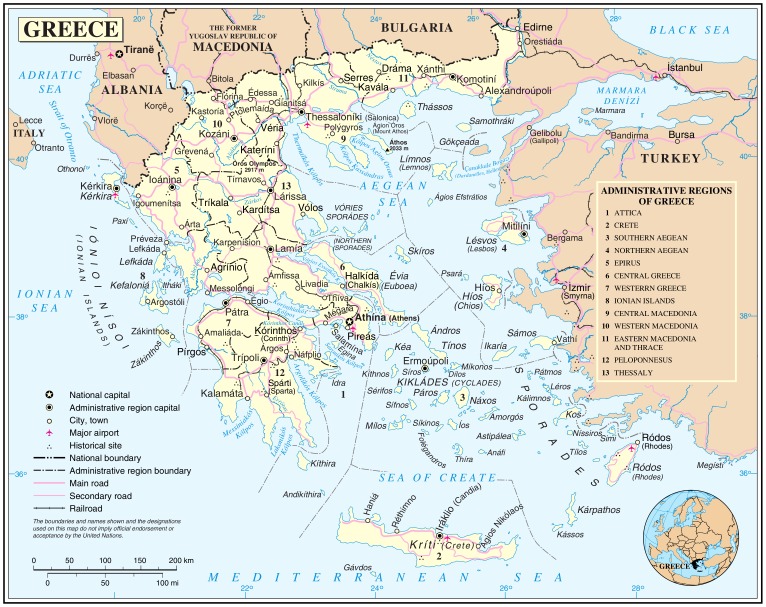
Map of Greece, http://www.worldofmaps.net/uploads/pics/generelle-karte-griechenland.png **, accessed July 18, 2012.**

Greece represents an intriguing location for such a center of excellence (Figure 1). This nation is centrally located between Eastern Europe, the Middle East, and North Africa and therefore is in a position to undertake unique studies on the ecology, transmission, and epidemiology of the NTDs across the region. A recent example is the changing of ecology of brucellosis and other zoonoses with increasing transmission in the Balkan Peninsula [5], as well as the re-emergence of vivax malaria in Greece [6]–[8]. Greece has also become one of Europe's major gateways for immigrants arriving from Africa and the Middle East [9], [10]. Equally relevant is the devastating recent downturn in the Greek economy, including a stifling level of unemployment that is reaching 50% among young people together with dramatic cutbacks in government services and new austerity measures [11]. There are real concerns about the future of Greece's ability to maintain its previously robust scientific research infrastructure. Indeed, there have already been severe cuts to science budgets and salaries at leading Greek scientific institutions, with the anticipation of additional cuts and consolidations likely [12].

At the same time, there is every reason to anticipate an emergence or re-emergence of NTDs in Greece just as we have seen in other areas of economic collapse and post-conflict in Europe and the Middle East. European Union or private support of a Hellenic center of excellence for NTDs could help to fend off declines in Greek biomedical science and prevent a potential brain drain to northern Europe, Australia, and elsewhere, while simultaneously providing urgently needed research and development for a nation that for centuries has bridged the cultures of Europe, North Africa, and the Middle East. Such an NTD center could be established by expanding infrastructures in existing centers such as the Hellenic Pasteur Institute, which is conducting important research on leishmaniasis and other NTDs [13]; the Foundation of Research and Technology-Hellas (FORTH) [14]; or the Hellenic Center for Disease Control and Prevention (HCDCP) [15], among others. It is important to point out that such a program should occur within the context of overall increases in financial support for Greek scientific institutions, and possibly even European Union funding.

A Hellenic center for NTDs is not a panacea, but it would be a welcome and potentially transformative institution that would address a pressing neglected disease problem linked to poverty and destabilization in the region. It could also provide urgently needed research and possibly new antipoverty drugs, diagnostics, and vaccines benefiting impoverished people and their animals everywhere. Such a center could promote global, neglected disease research much as other pan-European institutes in Germany (EMBO - European Molecular Biology Organization), Switzerland (CERN – European Center for Nuclear Research), and Italy (ICTP – The Abdus Salam International Centre for Theoretical Physics) have promoted outstanding studies in molecular biology, physics, and other key scientific areas. A Greek center for NTDs would go a long way to fostering urgently needed research and reducing poverty and disease.
